# Comparing the Scalpel-Bougie-Tube Emergency Front-of-Neck Airway (eFONA) Technique on Conventional Manikins and Ovine Larynges: Evaluating Cost, Realism, and Performance in Anaesthetic Trainees

**DOI:** 10.7759/cureus.40040

**Published:** 2023-06-06

**Authors:** Ahmed Abdelhamid, Sadhana Sapra

**Affiliations:** 1 Anaesthesiology, Walsall Manor Hospital, Walsall, GBR

**Keywords:** simulation-based training, airway management training, cricothyrotomy, medical education, anaesthesia novices, scalpel bougie tube, ovine, front-of-neck airway, cannot intubate cannot oxygenate

## Abstract

Background

Emergency front-of-neck airway (eFONA) is a crucial life-saving procedure in "cannot intubate, cannot oxygenate" (CICO) situations. It is essential to teach and maintain eFONA skills for healthcare providers, especially anesthesiologists. This study aims to assess the effectiveness of cost-effective ovine larynx models compared to conventional manikins in teaching eFONA using the scalpel-bougie-tube technique to a group of anaesthesia novices and newly appointed anaesthetic Fellows.

Methods and study design

The study was conducted at Walsall Manor Hospital, a district general hospital in the Midlands, UK. Participants underwent a pre-survey to assess familiarity with FONA and the ability to perform a laryngeal handshake. After a lecture and demonstration, participants performed two consecutive emergency cricothyrotomies on both ovine models and conventional manikins, followed by a post-survey to assess their confidence in performing eFONA and rate their experience using sheep larynges.

Results

The training session significantly improved the participants' ability to perform a laryngeal handshake and their confidence in performing eFONA. The majority of participants rated the ovine model higher in terms of realism, difficulty with penetration, difficulty in recognising landmarks, and difficulty in performing the procedure. Additionally, the ovine model was more cost-effective compared to conventional manikins.

Conclusion

Ovine models provide a more realistic and cost-effective alternative to conventional manikins for teaching eFONA using the scalpel-bougie-tube technique. The use of these models in routine airway teaching enhances the practical skill set of anaesthesia novices and newly appointed anaesthetists, better preparing them for CICO situations. However, further training with objective assessment methods and larger samples is needed to corroborate these findings.

## Introduction

Emergency front-of-neck airway (eFONA) is a life-saving procedure for patients who cannot be intubated or oxygenated ("cannot intubate, cannot oxygenate" (CICO)). It is essential to identify these situations and perform eFONA before a hypoxic cardiac arrest.

The Difficult Airway Society (DAS) presented its guidelines for the management of unanticipated difficult tracheal intubation and recommended a scalpel-bougie-tube cricothyroidotomy technique as the first-line because it is the fastest and most reliable method of securing the airway in an emergency [1]. This requires "a laryngeal handshake" to identify the cricothyroid membrane, then a transverse stab incision through the cricothyroid membrane (or an 8-10 cm midline vertical incision if the cricothyroid membrane is impalpable), followed by sliding a bougie into the trachea and railroading a lubricated 6.0mm cuffed tracheal tube.

The frequency of CICO and eFONA situations is influenced by factors such as the healthcare provider's hospital flow, qualifications, and experience, as well as the patient's specific medical condition. Most medical personnel have little, if any, clinical experience performing eFONA [2].

The Fourth National Audit Project of the Royal College of Anaesthetists (NAP4) and the DAS recommended that "all anaesthetists must be trained in emergency cricothyroidotomy and keep their skills up to date" [3].

The need to obtain eFONA can be one of the most terrifying situations a clinician can experience, especially with no prior experience. And a real CICO situation includes a level of stress that is difficult to simulate in training. Moreover, conventional manikins do not simulate real-time physiological feedback with relevant high-stress levels for the provider.

## Materials and methods

The department of anesthesiology at Walsall Manor Hospital coordinates an Airway Day for newly appointed, qualified anaesthetists and anaesthesia trainees. This educational event encompasses training on Plan D strategies, including front-of-neck airway (FONA) procedures, to enhance the practical skill set of the participants. We compared performing the most advocated eFONA approach, the scalpel-bougie-tube technique, on conventional manikins and sheep larynges.

Our aim in this study is to 1) assess the ability to perform a laryngeal handshake to identify the cricothyroid membrane; 2) assess the ability to perform the FONA procedure; 3) rate the realism of the sheep model in terms of the reality of skin, difficulty with penetration, difficulty with landmark recognition, difficulty with the procedure, and overall experience; and 4) compare the cost for each attempt at the simulation.

Population

The participants (total number: 14) were a group of anaesthetic novices (n=4) and newly appointed international medical graduates with different levels of experience. Two participants with one to three years of experience, two with three to five years of experience, and six with more than five years of experience were included in the study. Trainees who had performed a cricothyroidotomy on a patient were excluded.

Study protocol

**Figure 1:**
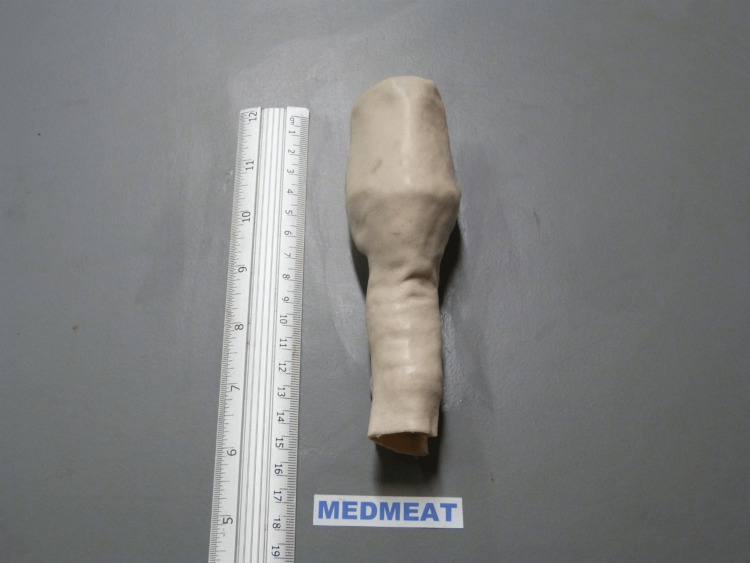
Ovine larynx with skin

Sheep larynx models were supplied by a reputable wet lab supplier with proper storage and handling (Wetlab Ltd., Warwickshire, UK) (Figure 1).

**Figure 2:**
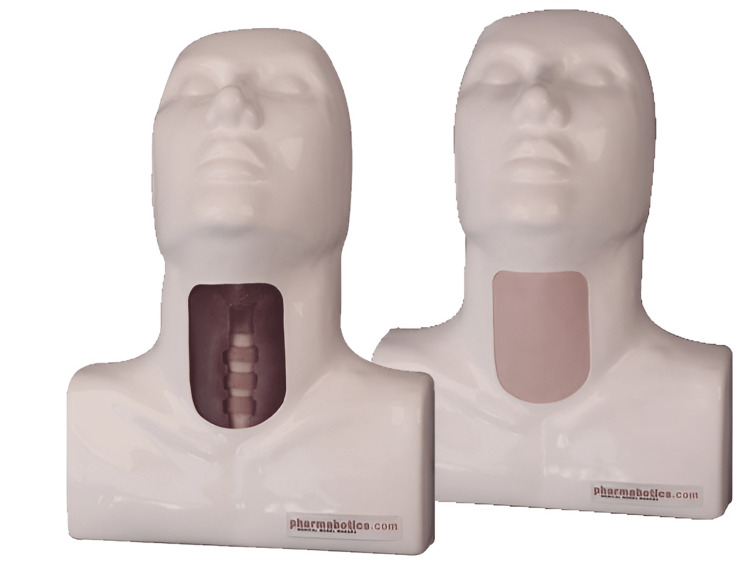
CYT100 Cricotracheotomy Trainer by Pharmabotics Ltd, UK

The tissues were a by-product derived from healthy animals and intended for human consumption. The sheep were slaughtered in European Economic Community (EEC)-approved abattoirs in accordance with all EEC regulations. Therefore, no ethics committee approval was needed. Conventional cricotracheotomy trainers (CYT100 Cricotracheotomy Trainer by Pharmabotics Ltd., UK) (Figure 2) were used for comparison.

A pre-survey was used to assess participants' familiarity with FONA and their ability to perform a laryngeal handshake.

**Figure 3:**
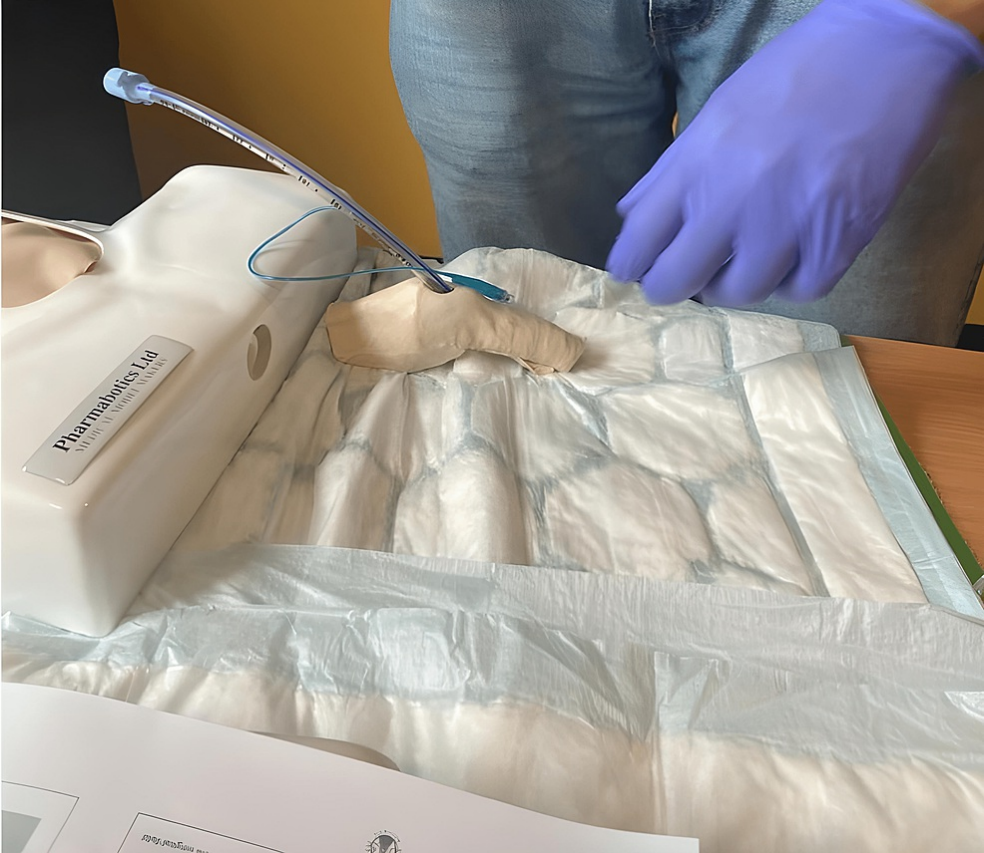
Training using the proposed ovine tissue

After a lecture and a demonstration, each participant performed two consecutive emergency cricothyrotomies on both models, followed by a post-survey to assess confidence in performing FONA in a CICO situation and rate the experience of using sheep larynges in the simulation (Figure 3).

Participants were asked to assign a score between one and five, with each point representing the achievement of each of the following characteristics: realism of the skin, difficulty with penetration, difficulty in recognising landmarks, and difficulty in performing the procedure.

A score of one indicated no difference compared to a conventional manikin, while a score of five represented the achievement of all four criteria.

## Results

Participants' ability to perform a laryngeal handshake

**Figure 4:**
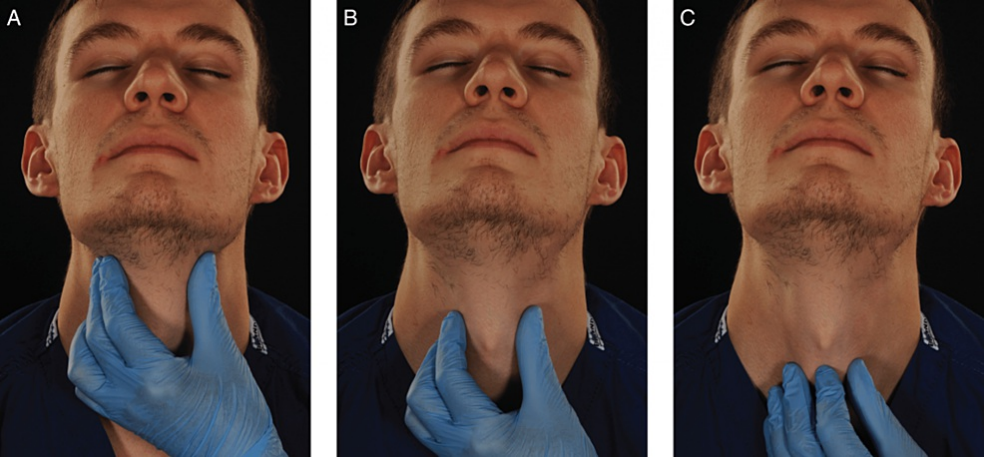
The laryngeal handshake as described in the DAS guidelines (A) Using the non-dominant hand to identify the hyoid and thyroid laminae, (B) stabilising the larynx between the thumb and middle finger, and (C) sliding down the neck to locate the cricothyroid membrane with the index finger Image courtesy: DAS Guidelines, 2015 [1]

A cross-tabulation of the assessment of the participants' ability to perform a laryngeal handshake before and after the session is seen in Table 1 and Figure 4.

**Table 1:** Cross-tabulation of the participants' ability to perform a laryngeal handshake

Period	Yes	No
Before the session	4	10
After the session	14	0

There were 10 participants who were not able to perform the handshake before the session. After a one-to-one hands-on teaching session, they recorded that they were able to palpate the cricothyroid membrane more easily using the technique, giving strong evidence of the effectiveness of the training.

Participants' ability to perform FONA

To compare the two groups of "before session" and "after session," we went for non-parametric analysis (Wilcoxon sign-ranked test) (Tables 2-3) using IBM Statistical Package for Social Sciences (SPSS) Version 26.0 for Windows (IBM, New York, U.S.) as our data were non-normally distributed.

**Table 2:** Descriptive statistics of confidence in performing FONA before and after the session

	N	Mean	Standard deviation	Percentiles		
				25th	50th (Median)	75th
Familiarity with the FONA concept before the session	14	3.07	.997	2.00	3.00	4.00
Confidence in performing FONA after the session	14	4.14	.949	3.00	4.50	5.00

**Table 3:** Wilcoxon signed-rank test: (A) the ability to perform FONA is worse; (B) improved confidence in performing FONA; (C) no change

		N	Mean rank	Sum of ranks
Performing FONA before and after the session	Negative ranks (A)	1	5.00	5.00
	Positive ranks (B)	11	6.64	73.00
	Ties (C)	2		
	Total	14		

The test showed that the simulation session elicits a statistically significant median change in the learning experience, making healthcare providers better prepared to handle an airway emergency in a clinical setting (z = -2.801, p = .005) (Table 4).

**Table 4:** Related-samples Wilcoxon signed-rank test summary

Test statistics	73.000
Standard error	12.140
Standardised test statistic	-2.801
Asymptotic significance (2-sided test)	.005

The reality of the model

The majority of participants (n=11) assigned a score of five to the tissue model, indicating that it met all four criteria and provided a more realistic experience, while the rest (n=3) scored their experience four.

## Discussion

A cricothyrotomy may be a life-saving procedure in a CICO situation [4,5]. Cricothyrotomy is rarely encountered in the clinical setting, especially in a small district general hospital, making alternative methods to teach this high-stakes procedure crucial [6]. The Fourth National Survey in the United Kingdom reported that the failure rate of cricothyrotomy performed by anaesthetists was as high as 64% [7]. It is essential to choose a suitable cricothyrotomy practice model that will help improve the success rate of anaesthetists in performing cricothyrotomy in emergencies and effectively improve the success rate of emergency airway rescue [8].

There are many models for airway management training, including manikins, animals, cadavers, volunteers, newly dead subjects, and live patients [9-10]. Wang et al. (2007) presented an innovative and inexpensive method for teaching cricothyrotomy using lamb and veal trachea [11]. However, it had its drawbacks, such as using synthetic skin, and the lamb and veal tracheas were at least 50% larger than humans. Hughes et al. (2018) developed a cricothyrotomy trainer using a fused deposition modelling (FDM) three-dimensional (3D) printer and innovative bleeding tissue to enhance fidelity [12]. Zagona-Prizio et al. (2023) found that bronchoscopy-enhanced cricothyrotomy training in cadavers is feasible and enhances the training experience for non-surgeon providers [13]. Not to mention, the use of a porcine trachea model for cricothyrotomy training is well established [5]. However, we could not find studies assessing the practicality of using ovine models in training the scalpel-bougie-tube technique in CICO teaching.

This study compared the usefulness and practicality of ovine models and conventional manikins in scalpel bougie tube eFONA in routine airway teaching in a small DGH. Skin-covered ovine models were rated higher by most participants in terms of the realism of the skin, difficulty with penetration, difficulty in recognising landmarks, and difficulty in performing the procedure. Another advantage was the low cost of the ovine model at £7.5 compared to £507 for the conventional model, with an additional £105 for the skin replacement for the manikin that needs to be changed every four attempts on average, bringing the cost to at least £25 per attempt.

When using an animal model for skill training, there is a concern about zoonosis, which is the transmission of infectious diseases from animals to humans. The tissue supplier needs to be a registered transporter of animal by-products and approved by the Animal and Plant Health Agency, which makes them legally able to collect and deliver animal tissue for medical or educational purposes [14-15].

Limitations of the study were: 1) it can be challenging to simulate patients with a high BMI or trauma patients where bleeding might be present; 2) the study was done using self-evaluation, so a more objective assessment is needed; and 3) participants were limited to two attempts at eFONA due to time constraints.

## Conclusions

The use of ovine models in scalpel-bougie-tube eFONA training provides a more realistic and cost-effective alternative to conventional manikins for routine airway teaching in a small district general hospital (DGH) setting. Participants reported improved confidence in performing the laryngeal handshake and the FONA procedure after the training session. The ovine model's increased realism in terms of skin, penetration, landmark recognition, and procedural difficulty contributes to a more comprehensive learning experience for anaesthesia novices and newly appointed anaesthetists. Although some limitations exist, such as the challenge of simulating high BMI or trauma patients and the reliance on self-evaluation, the ovine model remains a valuable and practical addition to airway management training.
